# Incidence, Clinical Characteristics, and Underreporting of Low Back Pain in Physically Active Pregnant Women: Prospective Cohort Study

**DOI:** 10.3390/medicina62010061

**Published:** 2025-12-28

**Authors:** Luz M. Gallo-Galán, José L. Gallo-Vallejo, Juan Mozas-Moreno

**Affiliations:** 1 Ginefiv, 28703 Madrid, Spain; luzma.gallo.galan@gmail.com; 2 Department of Obstetrics and Gynecology, University of Granada,18016 Granada, Spain; joselgallov@ugr.es; 3 Service of Obstetrics and Gynecology, Virgen de las Nieves University Hospital, 18014 Granada, Spain; 4 CIBER Epidemiología y Salud Pública (CIBERESP), Instituto de Salud Carlos III (ISCIII), 28019 Madrid, Spain; 5 Instituto de Investigación Biosanitaria de Granada (ibs.GRANADA), 18014 Granada, Spain

**Keywords:** low back pain, pregnancy, exercise, incidence, primary health care

## Abstract

*Background and Objectives:* Low back pain (LBP) is one of the most frequent complications during pregnancy, with a high and variable incidence. LBP has been associated with physical inactivity, but it has not been evaluated exclusively in physically active (PA) pregnant women. This study aimed T to estimate the incidence of LBP in PA pregnant women and describe its clinical, functional, emotional, and occupational impact. *Materials and Methods:* A prospective cohort of 147 women with PA pregnancies was recruited between gestational weeks 11 and 13^+6^. Most (92.5%) hold a university degree. All received standardized informational intervention based on international recommendations on PA during pregnancy and LBP prevention. Data were collected through an in-person interview in the first trimester and a postpartum follow-up phone interview. PA was assessed using the International Physical Activity Questionnaire (IPAQ, short version), and LBP intensity was evaluated using the Visual Analog Scale (VAS). *Results:* LBP occurred in 64.6% of participants, despite maintaining regular PA. Pain intensity was higher in standing position (VAS = 4.9) and lower in lateral decubitus (VAS = 2.7). More than half (55.8%) did not seek medical consultation. LBP was associated with functional limitations (work, sleep, walking), emotional distress (52.6%), and work leave (30.5%; mean 9.4 weeks). In the multivariable logistic regression analysis, standing occupational position showed a borderline association with LBP (OR = 2.14; 95% CI: 1.00–4.55; *p* = 0.047), while a history of LBP in a previous pregnancy showed a statistically significant association (OR = 2.89; 95% CI: 1.12–7.48; *p* = 0.029). Higher PA levels during pregnancy were associated with slightly lower odds of LBP (OR = 0.91 per 500 MET·min/week; 95% CI: 0.83–0.99; *p* = 0.032), although the magnitude of this association was small. *Conclusions:* LBP showed a high incidence even among PA and highly educated pregnant women. More than half of the women did not seek medical consultation, suggesting potential under-recognition of LBP. Standing occupational position and a previous pregnancy-related LBP were identified as independent risk factors associated with LBP in the multivariable model. Higher PA levels were inversely associated with LBP.

## 1. Introduction

Low back pain (LBP) is a very common condition during pregnancy, with a global prevalence ranging from 13.2% [1] to 81% [2], depending on the characteristics of the populations studied. This prevalence tends to increase as pregnancy progresses [3,4,5], reaching up to 85.5% in the third trimester [6]. Pregnancy-related LBP can significantly compromise maternal functionality, interfering with daily activities such as walking, working, or sleeping, and negatively affecting mood and quality of life. Its socioeconomic impact has also been documented, mainly through increased work absenteeism [4,5,6,7]. Despite its high prevalence, more than 50% of pregnant women with LBP receive little or no specific medical care, partly due to the mistaken perception that LBP is a physiological and inevitable consequence of pregnancy [8]. This misconception may discourage regular physical activity (PA), favoring sedentary behavior during gestation [9], which has been associated with a higher risk of hypertensive disorders of pregnancy, including gestational hypertension and preeclampsia [10], as well as an increased rate of cesarean deliveries [10,11].

Several international guidelines recognize exercise as a key preventive and therapeutic strategy for pregnancy-related LBP. The Canadian Guideline for Physical Activity throughout Pregnancy highlights that regular exercise is associated with reduced LBP severity [12]. Likewise, the American College of Obstetricians and Gynecologists recommends strengthening abdominal and back muscles and performing water-based exercise [13], while the Royal Australian and New Zealand College of Obstetricians and Gynecologists also emphasizes that regular exercise can help reduce LBP during pregnancy [14]. Other evidence-based guidelines, including those from the Australian Government Department of Health [15] and Sports Medicine Australia [16], specifically support aquatic programs for preventing and relieving LBP. Collectively, these recommendations underline the importance of integrating regular PA into prenatal care, given its broad maternal–fetal health benefits and its proven efficacy in preventing and alleviating LBP. Empirical evidence further reinforces this consensus, showing that exercise exerts a protective effect against LBP and significantly reduces its occurrence [17].

Despite the robust evidence supporting the benefits of PA on pregnancy-related LBP, no studies have been identified that standardize PA assessment among pregnant women with LBP using objective units such as MET·min/week. In previous research, PA has often been recorded dichotomously (“exercise during pregnancy: yes/no”) [17,18], or defined using criteria based on sessions lasting ≥10 min at least twice per week, without conversion to MET·min/week [19]. Consequently, the absence of precise quantification and validated criteria limits comparability between studies and hinders the ability to establish an accurate dose–response relationship between PA and LBP during pregnancy. Available evidence consistently indicates that gestational LBP is associated with low levels of PA, whereas regular exercise is linked to a lower frequency and intensity of pain [20,21,22]. However, this association has not been evaluated exclusively in PA pregnant women, as most studies have focused on populations not stratified by activity level.

In this context, the objectives of the present study were to determine the incidence of LBP in a cohort of PA pregnant women with uncomplicated pregnancies, to characterize its clinical manifestations, to analyze its impact on functional, emotional, and occupational domains, and to identify independent factors associated with the presence of LBP during pregnancy.

## 2. Materials and Methods

### 2.1. Study Design and Setting

A prospective cohort study was conducted at La Moraleja University Hospital (Madrid, Spain). The cohort consisted of pregnant women who were advised to engage in regular physical exercise throughout pregnancy. Participants were followed longitudinally during gestation to determine the incidence of LBP at different stages of pregnancy. Participants were recruited from the specialized first-trimester clinic for low-risk pregnancies, including only those women who attended between 11 and 13^+6^ weeks of gestation. The study was carried out between October 2023 and June 2025, comprising two distinct phases: the first phase extended from October 2023 to September 2024, and the second phase concluded in June 2025. Although recruitment and follow-up during pregnancy were prospective, most outcome variables, including PA during pregnancy and all LBP-related measures, were collected postpartum, giving the study a partially retrospective component. All women participating in the study received both verbal and written information regarding the purpose of the research and were informed of their right to withdraw consent at any time during the study. The study was approved by the Research Ethics Committee of La Princesa University Hospital (Madrid, Spain) (No. 5365).

Study Population
Inclusion Criteria

Pregnant women were considered eligible if they met the following criteria: maternal age of 18 years or older; singleton, uncomplicated, physiologically normal pregnancy; PA prior to pregnancy and up to recruitment; and attendance at the protocolized first-trimester visit intended for ultrasound examination and corresponding obstetric follow-up. Participants were classified as PA during pregnancy according to the World Health Organization (WHO) Guidelines on Physical Activity and Sedentary Behavior for pregnant women, which recommend engaging in at least 150 min of moderate–intensity aerobic activity per week, or an equivalent combination of moderate and vigorous-intensity activity [23]. This corresponds to a minimum of 600 MET·min/week, as assessed during the postpartum interview using the short-form International Physical Activity Questionnaire (IPAQ) [24].

Exclusion Criteria

**Figure 1:**
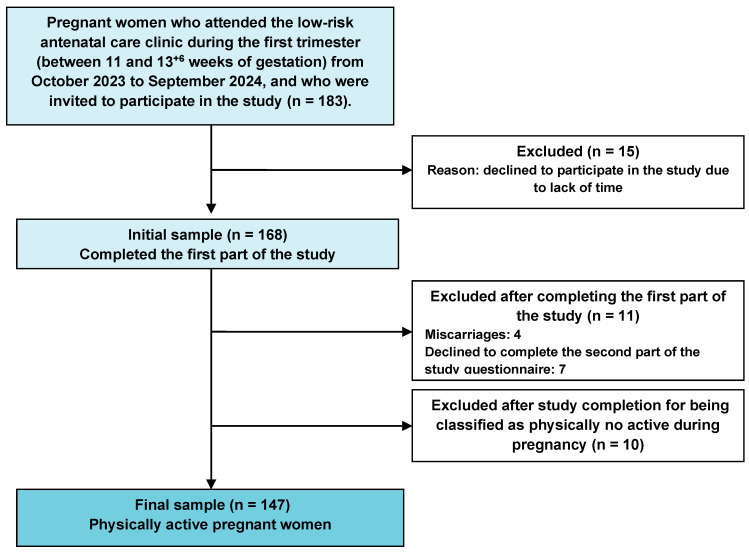
Flowchart of the selection, inclusion, and follow-up process of the study participants. The exclusion criteria and the final number of pregnant women who completed each phase of the study are shown.

Women were excluded if they presented with a diagnosis of obstetric or gestational pathology at the time of enrolment, or with chronic or acute diseases (musculoskeletal, cardiovascular, neurological, or other conditions) that limited or contraindicated the safe practice of physical exercise during pregnancy. Additionally, women who did not meet the WHO criteria for being PA during pregnancy (i.e., less than 600 MET·min/week or under 150 min of moderate-intensity activity per week) were excluded, ensuring that the study cohort comprised exclusively active participants. The flow of participants through the study, including recruitment, exclusion criteria, and follow-up, is shown in Figure 1.

### 2.2. Instruments

Participants were provided with a detailed information dossier that included: the scientifically evidenced benefits of physical exercise during pregnancy for maternal, fetal, neonatal, and infant health; recommendations regarding the duration, frequency, intensity and type of PA; a description of the absolute and relative contraindications for exercise; warning signs indicating the need to discontinue exercise; and, finally, guidance on activities to be avoided and strategies to prevent or relieve LBP during gestation.

Questionnaires

Data for this study were collected using an ad hoc questionnaire. During a face-to-face interview conducted in the first trimester of pregnancy, at the time of recruitment, general information about the participants was obtained, including identification data, sociodemographic variables, relevant medical history, lifestyle factors, obstetric history, and anthropometric parameters. Because no standardized or validated international or Spanish questionnaire exists for assessing several of the clinical and contextual variables included in this study, an ad hoc instrument was specifically developed for this research. Its content was designed and reviewed by clinical experts in obstetrics and researchers with experience in questionnaire development to ensure conceptual adequacy, clarity, and relevance.

The ad hoc questionnaire was designed to provide a broad and detailed description of pregnancy-related LBP, including its onset, temporal pattern, duration, irradiation, aggravating factors, and perceived triggers. It also captured the functional impact of LBP on daily activities (work, sleep, walking), emotional repercussions, health care utilization, and the perceived effectiveness of different management strategies. The primary aim of this instrument was descriptive and exploratory, focusing on characterizing the clinical presentation and real-life impact of LBP during pregnancy. The questionnaire was not intended to generate diagnostic classifications or composite disability scores, nor to be used for diagnostic comparisons. Instead, it sought to offer a comprehensive clinical and contextual overview of pregnancy-related LBP, aligned with the exploratory nature and objectives of the study. For this reason, standardized disability indices such as the Oswestry Disability Index were not used. Although such instruments are widely applied in chronic LBP populations, they are primarily designed to quantify functional disability through a single summary score and are less suitable for capturing pregnancy-specific pain characteristics, temporal variability, contextual triggers, and obstetric-related functional limitations, which were central to the objectives of the present study.

In this same initial interview, PA performed during the six months preceding conception was assessed using the short version of the IPAQ, to estimate the type, frequency, duration, and intensity of habitual PA prior to gestation. This information was used to characterize baseline activity levels, to compare pre-pregnancy and gestational PA, and to explore the influence of pre-pregnancy PA on the incidence and intensity of LBP during pregnancy.

Following childbirth, a follow-up telephone interview was conducted to collect data on PA or exercise undertaken during pregnancy, again using the IPAQ. Detailed information was also obtained regarding the presence of LBP during pregnancy, through a block of 20 structured items that included variables related to location, frequency, duration, clinical characteristics of pain, triggering or aggravating factors, interventions used, and perceived efficacy. Because therapeutic advice regarding LBP was not formally standardized across providers and could differ between clinicians, these management-related data were collected descriptively but were not used as analytical variables. During this postpartum interview, participants also assessed the perceived usefulness of the information dossier and indicated whether it had influenced their motivation to remain PA; these data were analyzed but are not presented in detail, as they were ancillary to the main study objectives. The intensity of LBP was assessed using a 0–10 Visual Analog Scale (VAS), where 0 represented “no pain” and 10 “the worst pain imaginable.” Scores were recorded for four positions: sitting, standing, supine, and lateral decubitus. During the initial face-to-face interview, the numerical VAS was provided in printed form. During the postpartum telephone interview, the same scale was administered verbally, with all numerical options read aloud to participants to ensure equivalence across modes of administration. LBP was defined as pain located between the twelfth rib and the gluteal fold, with or without irradiation to the lower limbs.

### 2.3. Statistical Analysis

Quantitative variables were first examined for distributional properties using the Shapiro–Wilk test and visual inspection of histograms and Q–Q plots. Variables such as MET·min/week, sick-leave duration, and several VAS pain scores showed non-normal distributions. Normally distributed variables were summarized as mean, standard deviation (SD), and minimum and maximum values, while non-normal variables were described using median and interquartile range (IQR). Parametric tests (*t*-tests and ANOVA) were applied when distributional properties and sample size supported their robustness; parallel non-parametric analyses (Mann–Whitney U and Kruskal–Wallis tests) yielded consistent results. Categorical variables were summarized as absolute and relative frequencies (%). *p*-values reported for descriptive analyses were used strictly for exploratory purposes to identify non-random patterns.

Associations between qualitative variables and the presence of LBP were examined using Chi-squared (χ^2^) or Fisher’s exact tests when appropriate. Student’s *t*-test was used to compare means between two independent groups, and paired *t*-tests for intra-individual comparisons before and during pregnancy. ANOVA with Tukey’s post hoc test was applied for comparisons involving more than two groups. Effect sizes were estimated using η^2^ when applicable. Correlations between MET·min/week and VAS pain scores were assessed using Pearson coefficients and simple linear regression.

A multivariable logistic regression model was fitted to identify factors associated with LBP during pregnancy. Variables were selected based on (1) *p* < 0.10 in bivariate analysis or (2) clinical relevance supported by the literature (occupational posture, previous pregnancy-related LBP, physical activity). Adjusted odds ratios (OR) and 95% confidence intervals (95% CI) were calculated. Linearity of continuous predictors in the logit was assessed using graphical evaluation and Box–Tidwell transformations; no violations were observed for MET·min/week. Multicollinearity was evaluated using the Variance Inflation Factor (VIF); no relevant multicollinearity was detected among the predictors included in the final model. To minimize overfitting, the number of predictors was restricted according to contemporary events-per-variable recommendations. Model calibration and discrimination were assessed using the Hosmer–Lemeshow test and the area under the receiver operating characteristic (ROC) curve, respectively. Sample size estimation was based on an expected prevalence of LBP of 60%, with 95% confidence level and absolute precision of 7.5%, yielding a minimum required sample of 164 women. Statistical analyses were conducted using R software (version 4.3.2). A significance threshold of *p* < 0.05 was applied.

### 2.4. Sample Characteristics

The final sample consisted of 147 women with physiologically normal pregnancies, all of whom were classified as PA during pregnancy according to WHO criteria. Their mean energy expenditure was 1896.7 ± 1012.1 MET·min/week, with values ranging from 660.0 to 6597.0 MET·min/week and percentile distribution P25 = 1229.5, P50 = 1746.0, and P75 = 2390.0 MET·min/week. The mean age of participants was 34.1 ± 4.0 years, with an average height of 163.9 ± 5.7 cm and a mean pre-pregnancy weight of 66.7 ± 9.8 kg. Regarding parity, 66.7% (n = 98) were primiparous and 33.3% (n = 49) multiparous. Most participants (92.5%) held a university degree, while 7.5% had completed secondary education. From an occupational perspective, 97.3% of the women were engaged in paid employment outside the home, of whom 83.0% worked full-time and 8.2% part-time; the remainder were unemployed, on sick leave, on career break, or dedicated exclusively to domestic tasks. The average working day was 7.7 h (range: 4–12 h). Regarding working posture, 55.1% of participants remained mostly seated, 30.6% alternated between postures (mixed), and 14.3% remained preominantly standing.

## 3. Results

When comparing groups according to the presence of LBP, women who reported this symptom had a significantly lower mean age (33.5 ± 4.2 years) than those without LBP (35.2 ± 3.6 years) (*p* = 0.011). Regarding the incidence of LBP, 64.6% (n = 95) of participants reported experiencing this symptom during the current pregnancy, whereas 35.4% (n = 52) did not. Among symptomatic women, the onset of LBP occurred most frequently during the third trimester (60.0%), followed by onset in both the second and third trimesters (22.1%) and onset restricted to the second trimester (11.6%). Only 4.2% reported pain throughout all three trimesters (*p* < 0.0001). The weekly frequency of LBP was predominantly >3 times per week (55.8%), followed by twice (22.1%), three times (11.6%), and once weekly (10.5%) (*p* < 0.001) (Table 1).

The time of day when LBP occurred, symptoms were most frequently reported during the daytime (42.1%), followed by nighttime (28.4%) and both day and night (17.9%) (*p* < 0.0001). Conversely, the highest pain intensity was reported in the afternoon (30.5%), followed by nighttime (27.4%) and afternoon/night (18.9%); morning pain was less common (11.6%) (*p* < 0.0001). With respect to the type of LBP, intermittent pain was most frequent (68.4%), compared with continuous pain (31.6%) (*p* < 0.001). In terms of duration, the acute form (<4 weeks) predominated (50.5%), followed by subacute (4–12 weeks) in 33.7% of cases and chronic (>12 weeks) in 15.8% (*p* < 0.001). LBP irradiation was reported by 55.8% of symptomatic women, while 44.2% reported no irradiation (*p* = ns). Among those with irradiation, the most frequently affected region was the gluteal area (35.8%), followed by the leg (24.5%) and the hip (15.1%), with a significantly non-homogeneous distribution (*p* < 0.001). Regarding LBP intensity measured using the VAS, the highest mean scores were observed in the standing (4.9 ± 2.1) and sitting (4.6 ± 2.1) positions, followed by the supine position (3.9 ± 2.5), values corresponding to the moderate pain range. The lowest intensity was reported in the lateral decubitus position (2.7 ± 2.2). These differences were statistically significant (*p* < 0.001). Although some variables showed skewed distributions, non-parametric tests confirmed the same direction and significance of the findings. When analyzing the relationship between PA during pregnancy (MET·min/week) and LBP intensity in different positions (sitting, standing, supine, lateral), very weak negative associations were observed, none of which reached statistical significance. Simple linear regression models confirmed these findings, with R^2^ values close to zero (Table 2).

The activities that most aggravated LBP were static postures, mainly sitting (37.9%) and standing (28.4%) (*p* < 0.001). The daily activities most affected by LBP were work (26.3%), sleep (16.8%), and walking (14.7%). Only 5.3% reported having experienced no functional limitations (*p* < 0.001). Strategies considered effective for relieving LBP included physical exercise, which was the most frequently mentioned, either alone (33.7%) or in combination with massage, rest, or physiotherapy. Rest, either as a single or complementary measure, was reported as the second most common strategy (*p* < 0.001). With respect to emotional impact, 52.6% of participants stated that LBP had negatively affected their mood, while 47.4% reported no emotional repercussions. In the occupational domain, 30.5% of pregnant women reported having interrupted their professional activity due to LBP, whereas 69.5% continued working despite the pain (*p* < 0.001). Among those who discontinued work, the mean duration of sick leave was 9.4 ± 6.7 weeks, ranging from 0.5 to 28 weeks (Table 3).

Concerning medical consultations for LBP (Table 4), 44.2% (n = 42) of pregnant women with LBP sought medical advice, while 55.8% (n = 53) did not. Among those who did consult, 90.5% (n = 38) received a therapeutic recommendation, mainly physical exercise (57.1%), followed by pharmacological treatment (14.3%). In four cases (9.5%) no treatment was prescribed (*p* < 0.001 in both cases).

In the multivariable logistic regression analysis (Table 5), standing occupational position was significantly associated with a higher likelihood of LBP during pregnancy (OR = 2.14; 95% CI: 1.00–4.55; *p* = 0.047), compared with women who predominantly worked in a sitting position, although the confidence interval included 1.00, indicating borderline statistical significance and warranting cautious interpretation. PA during pregnancy was inversely associated with the presence of LBP (OR = 0.91 per 500 MET·min/week; 95% CI: 0.83–0.99; *p* = 0.032), but the magnitude of this association was small and should not be interpreted as a clinically meaningful protective effect. A history of LBP in a previous pregnancy was also associated with a higher likelihood of LBP in the current gestation (OR = 2.89; 95% CI: 1.12–7.48; *p* = 0.029), although, as with the other predictors, this represents an observational association rather than a causal effect. The model demonstrated good discriminative ability (AUC = 0.79) and adequate calibration (Hosmer–Lemeshow *p* = 0.56), and no relevant multicollinearity was detected among the predictors retained in the final model, consistent with the VIF analysis showing low values only for these variables.

## 4. Discussion

In our cohort of PA pregnant women, most of whom had a university education (92.5%) and received standardized, evidence-based informational counseling on exercise during pregnancy and LBP prevention, a high incidence of LBP was identified (64.6%). Most cases were of mild to moderate intensity but had significant functional, emotional, and occupational repercussions. Despite this high incidence, marked under-consultation was evident, as more than half of participants (55.8%) did not seek medical attention for this condition. This study was conducted in a cohort of pregnant women with specific characteristics that must be considered when interpreting the results. First, all participants were classified as PA according to the WHO criteria [23]. Second, the educational level was predominantly university (92.5%), much higher than that reported in other populations, where proportions of 23.8% [25] and 48.3% have been described [19]. This unique sociodemographic profile should be considered when interpreting the findings, as it differs substantially from that of broader obstetric populations. Furthermore, all participants received during the first trimester an evidence-based informational dossier on physical exercise and preventive strategies for LBP.

One of the most relevant findings was the low rate of medical consultation: only 44.2% attended clinical care, despite having an active profile, a high educational level, and access to information from early pregnancy. This finding aligns with previous literature documenting high rates of underconsultation among pregnant women with LBP. In a U.S. cohort, only 32% of women reported this symptom during prenatal check-ups, and only one-quarter received any therapeutic recommendation [26]. Similarly, other studies have reported that many pregnant women with LBP do not consult health professionals during pregnancy [27]. This study adds a novel contribution by identifying the level of care involved: primary care. In this setting, most women who consulted received therapeutic recommendations, mainly exercise, confirming that access to medical care translates into effective interventions. Previous research has suggested that cultural beliefs, normalization of LBP during pregnancy, or attitudinal factors may contribute to underconsultation [8,28], but these aspects were not evaluated in our study and therefore cannot be assumed to explain the patterns observed in this cohort. Furthermore, a recent review concluded that purely educational interventions are insufficient to reduce LBP during pregnancy and should be complemented by active strategies such as exercise to achieve clinically meaningful effects [29]. However, this evidence provides general context and cannot be used to infer the reasons for the underconsultation observed in our sample. Consequently, no conclusions can be drawn regarding the reasons underlying the low consultation rate, which should be explored in future research. Among women who did seek care, 90.5% received some form of therapeutic recommendation, with physical exercise being the most common (57.1%). This finding indicates that medical consultation results in beneficial interventions, reinforcing the importance of facilitating timely access to such resources from the early stages of pregnancy. Although prior studies have shown that lower educational level is associated with greater risk of moderate or severe LBP [30] and higher pain intensity [31], these findings cannot be extrapolated to interpret consultation behaviors in our sample, as these determinants were not assessed.

Another relevant finding of this study, considering the specific characteristics of the cohort, was the high incidence of LBP during pregnancy, reaching 64.6%. Despite this value falling within the broad range reported in the literature (13.2–81%) [5], most studies describe approximate prevalence values around 50% [32]. Therefore, the high incidence observed is noteworthy. Although our study did not include a comparison group of inactive women, the finding is particularly striking because, given the clinical and sociodemographic profile of our participants, highly educated and PA, a lower frequency of LBP would have been expected a priori, as these characteristics are traditionally considered potentially protective against this musculoskeletal condition. The discrepancy between expectation and findings may be related to methodological heterogeneity across studies, differences in definitions, timing of assessment, and instruments used for pain measurement. Likewise, population-specific characteristics such as educational level, occupation, or access to health services may influence the perception and reporting of symptoms. Finally, the absence of a standardized definition of pregnancy-related LBP contributes to variability in reported prevalence.

The high incidence of LBP in our cohort challenges the notion that PA alone can prevent its onset. The objective quantification of PA level using METs represents a relevant methodological contribution, as it allows confirmation that this high incidence occurs even among women classified as PA. Although the literature has extensively documented the high frequency of LBP during gestation, few studies have evaluated PA levels in a standardized manner. Most have addressed PA in a general way, without precise quantification or application of objective criteria for classification. Some studies have identified sedentary behavior or physically demanding jobs as risk factors for LBP [33,34,35]. Even in studies including PA women, standardized units such as MET·min/week or validated cut-off points for defining activity levels are rarely used. Thus, many lifestyle studies fail to precisely quantify exercise or to apply standardized criteria [26,36]. In our cohort, all participants reached that threshold, making direct comparison with classical prevalence studies difficult. Nevertheless, the results suggest that although PA may not necessarily prevent the occurrence of LBP, women in this cohort reported pain levels within the mild-to-moderate range, with lower values in the lateral decubitus position. This observation is consistent with a recent review identifying physical exercise as an effective strategy for reducing LBP intensity in pregnant women, particularly highlighting Pilates and aquatic exercise as safe and effective options [37]. Complementarily, it has been shown that sedentary pregnant women have a 30% higher likelihood of experiencing LBP or pelvic pain of greater intensity compared with active women, regardless of trimester or weight gain criteria [21]. Taken together, these findings suggest that PA may be helpful for symptom management during pregnancy, although it cannot be considered a definitive preventive strategy.

In relation to maternal age, the possible relationship between age and the onset of LBP has been analyzed with variable results. In our study, a significant trend toward higher LBP frequency was observed among younger women, in line with previous research [19,26,36]. Consistently, a recent systematic review confirmed a higher prevalence of LBP among younger pregnant women, supporting the hypothesis of increased vulnerability in this age group [38].

With respect to the timing of LBP onset, the highest frequency in our cohort was recorded during the third trimester (60.0%). This finding partially aligns with the results of Berber and Satılmış [6], who also observed an increase in LBP during this period, although with a higher prevalence (85.5%). However, recent studies have reported a more homogeneous distribution between the second (62%) and third trimesters (63%), suggesting that the progression of LBP throughout pregnancy may vary according to individual, biomechanical, and contextual factors [38]. Complementarily, the systematic review and meta-analysis by Salari et al. provided the strongest evidence of global prevalence trends, demonstrating a progressive increase throughout gestation: 28.3% in the first trimester, 36.8% in the second, and 47.8% in the third [5]. Our results are consistent with this ascending trend, although they differ from other studies reporting a higher frequency in the second trimester (43.9%) compared with the third (21.2%) [39]. These discrepancies reflect methodological heterogeneity and the influence of population characteristics on the temporal pattern of LBP occurrence. Overall, these data reinforce the need for uniform diagnostic criteria and standardized measurement tools to identify critical periods for clinical intervention and preventive follow-up during pregnancy.

Regarding clinical history, the presence of LBP in previous pregnancies was identified in our study as a significant predictive factor for recurrence in the current gestation. Among multiparous women, 51.0% reported having experienced LBP in a prior pregnancy, while 55.1% reported recurrence during the current one. These results are consistent with previous evidence demonstrating a strong association between a history of LBP in past pregnancies and its persistence in subsequent gestations [33,40]. Similarly, the history of LBP in previous pregnancies has been shown to be an independent risk factor for its occurrence in subsequent ones [41]. Other studies have confirmed this finding, identifying previous LBP as the main predictor of recurrence [17]. A significant correlation has also been observed between current and past gestational LBP, reinforcing the clinical importance of this obstetric history [19]. Taken together, these findings underscore the need to include LBP history as part of routine assessments in pregnant women and to design specific preventive strategies for those with prior episodes.

In terms of LBP pattern, intermittent pain predominated in our cohort (68.4%), surpassing continuous pain (31.6%). This suggests that, in most cases, gestational LBP fluctuates throughout the day or in response to specific postures or activities. Similar findings were reported by Carvalho et al., who observed an even greater proportion of intermittent pain (96.9%) compared to continuous pain (3.0%) [39]. The predominance of an intermittent pattern is consistent with the notion that gestational LBP often varies in relation to posture or daily activities, although the specific mechanisms involved were not assessed in this study. This distinction has clinical relevance, as it helps guide therapeutic strategies focusing on postural modification, ergonomics, and muscular strengthening. About LBP intensity, participants reported mild-to-moderate pain levels, with mean VAS scores of 4.9 while standing, 4.6 in sitting, 3.9 in supine, and 2.7 in lateral decubitus positions. These results fall within the range described in the literature [30,42]. The lower intensity observed in the lateral decubitus position is consistent with a reduced mechanical demand in this posture, although the underlying mechanisms were not evaluated. Furthermore, the fact that all participants were PA is consistent with studies reporting lower pain intensity among PA pregnant women [4,7].

Despite regular PA, a considerable proportion of participants reported functional limitations due to LBP, mainly affecting work (26.3%), sleep (16.8%), and walking (14.7%). These results confirm that LBP significantly impairs quality of life even among active women, consistent with evidence describing its negative impact on well-being, mood, and occupational performance [6,43]. Complementarily, other authors identified nighttime rest (49.6%), prolonged sitting (38.7%), and walking (37.2%) as the activities most related to the onset or exacerbation of LBP, partially coinciding with our findings [19]. Overall, the evidence suggests that posture-related activities, rest, and mobility function as key biomechanical factors in the development or aggravation of gestational LBP and should be addressed through personalized interventions focused on postural education, ergonomics, and therapeutic exercise. In our cohort, 52.6% of pregnant women reported that LBP negatively affected their mood, demonstrating emotional repercussions even in the absence of severe physical dysfunction. This finding is consistent with the literature describing a bidirectional association between gestational LBP and mood disorders, showing that women with LBP and pelvic pain have a higher risk of depression and anxiety [44]. Moreover, impaired mental health at the beginning of pregnancy has been associated with greater LBP intensity in later stages, confirming the interaction between physical and psychological components [45]. These results reinforce the need for a biopsychosocial approach to the assessment of gestational LBP, incorporating mood evaluation to optimize treatment and prevent the chronification of both physical and psychological discomfort during the perinatal period. Regarding occupational performance, 30.5% of participants in our cohort discontinued professional activity due to LBP, reflecting a clinically relevant functional impact. Although most continued working, this proportion indicates that LBP can justify temporary disability in a considerable number of cases. While the determinants of this decision were not assessed in our study, prior literature has described how workplace rigidity and reduced functional capacity may influence work interruption in pregnant women with LBP [46]. The results obtained fall within an intermediate range compared with other populations, where rates of sick leave due to LBP during pregnancy range between 28% and 68% [43,46]. These findings support the idea that, although frequent, LBP does not always lead to occupational incapacity, probably due to a combination of individual, occupational, and contextual factors that influence the decision to take medical leave. Several studies have identified pregestational LBP as a predictor of prolonged sick leave and university education as a protective factor [47], as well as demonstrating a dose–response relationship between pain intensity and absenteeism [30]. In our cohort, the mean duration of sick leave was 9.4 weeks (range 0.5–28), a value higher than that reported in Nordic settings, where the median duration was four days, with no significant differences between women performing unsupervised aquatic exercise and controls [3]. These differences could be explained by socio-occupational factors, job characteristics, and variability in disability policies. All together, these findings emphasize the importance of an individualized approach that considers not only pain severity but also its functional impact and the occupational context of each pregnant woman. However, although the multivariable logistic regression model showed adequate discrimination and calibration, the slightly smaller final sample size than initially estimated may have limited the statistical power of the analysis. Consequently, associations showing borderline statistical significance, such as occupational standing posture, should be interpreted with caution and confirmed in larger cohorts.

Among the main strengths of this study is its exclusive focus on a cohort of PA pregnant women, providing original data on LBP incidence in this specific obstetric subgroup. To date, no previous studies have analyzed LBP occurrence specifically in PA pregnant women, which gives this work a distinctive character and provides novel evidence in an underexplored area. This methodological approach also allowed a detailed exploration of the relationship between regular exercise and LBP occurrence in a homogeneous, clinically relevant group, reducing the influence of potential confounding factors related to sedentary lifestyle and comorbidities frequently present in broader population-based studies. Additionally, the sample was homogeneous, comprising women with physiological pregnancies, without obstetric risk factors, and with a high educational level (92.5% with university studies), which strengthens internal consistency but also limits external generalizability All participants received a structured informational intervention on exercise during pregnancy and LBP prevention, ensuring methodological coherence across the sample. Another notable strength is the use of internationally validated instruments, such as the short-form IPAQ to quantify PA in MET·min/week and the VAS for pain intensity, both of which enhance methodological robustness and comparability of results. Moreover, the study achieved a low attrition rate, increasing the internal validity of its findings. Finally, it provides unprecedented data on sick leave duration and medical consultation frequency for LBP in PA pregnant women, topics scarcely addressed in prior literature.

Nonetheless, this study presents several limitations that should be considered when interpreting the results. Although recruitment was prospective, information on LBP was obtained through a telephone interview conducted within the first postpartum month, introducing a retrospective component and the possibility of recall bias, particularly regarding the onset, frequency, and positional intensity of pain. To mitigate this limitation, participants were informed at recruitment that they would be contacted postpartum to report these outcomes, were familiarized in advance with the short version of the IPAQ and received a printed Visual Analog Scale (VAS), which was subsequently used as a reference during the interview. Despite these measures, recall bias cannot be completely excluded. Even the fact that previous literature suggests that maternal recall of pregnancy-related events may be reasonably reliable [48,49], this does not eliminate the potential for memory-related inaccuracies, even though participants were informed during pregnancy that this information would later be requested. The assessment of LBP throughout pregnancy may be a more comprehensive strategy than repeated reports. Furthermore, PA during pregnancy was assessed exclusively using the short-form IPAQ, a self-reported instrument that may overestimate moderate-to-vigorous PA and underestimate sedentary behavior compared with objective methods such as accelerometry. Although this questionnaire is widely used in pregnant populations [50] and has been validated in the Spanish population [51], it does not provide objective measurement. Objective tools such as accelerometry were not available in this study, due to logistical and funding constraints, which should be considered when interpreting the findings. Future studies should incorporate objective measurement tools when resources permit. Similarly, the ad hoc questionnaire used to collect clinical, functional, and emotional characteristics of LBP lacked formal validation in Spanish pregnant women, despite expert review to ensure content relevance. Another limitation is the homogeneity of the sample: 92.5% of participants had a university degree, all met the recommended PA criteria, and the study was conducted in a private university hospital. These characteristics may introduce selection bias and limit the generalizability of the findings to more diverse populations with different educational, socioeconomic, or physical activity profiles. While this homogeneity contributes to internal consistency, it simultaneously reduces external validity, and its dual nature has been considered both among the strengths and the limitations of the study. In addition, the study design did not include a comparative group of inactive women, which prevents causal inference and precludes evaluation of the relative role of regular physical activity in the occurrence of LBP; therefore, all interpretations must remain strictly observational. The final sample size (147 participants) was slightly smaller than the initially estimated sample (n = 164), which may have reduced the statistical power of the study and increased the risk of type II error, particularly for variables showing borderline statistical significance in the multivariable logistic regression model (e.g., standing occupational posture). Although this reduction resulted primarily from the strict application of predefined inclusion criteria, especially the requirement to meet PA thresholds during pregnancy, which could only be confirmed postpartum, it may have influenced the precision of some estimates, as reflected in wider confidence intervals. Nevertheless, the multivariable logistic regression model was constructed following conservative criteria to avoid overfitting, limiting the number of predictors according to events-per-variable recommendations, and demonstrated adequate discrimination and calibration.

Another limitation is the homogeneity of the sample: 92.5% of participants had a university degree, all met the recommended PA criteria, and the study was conducted in a private university hospital setting. These characteristics may introduce selection bias and limit the generalizability of the findings to more diverse populations with different educational, socioeconomic, or PA profiles. While this homogeneity contributes to internal consistency, it simultaneously reduces external validity.

In addition, the study design did not include a comparison group of inactive women, which prevents causal inference and precludes evaluation of the relative role of regular PA in the occurrence of LBP; therefore, all interpretations must remain strictly observational. Therapeutic recommendations reported by participants did not follow a standardized protocol and depended on individual clinical judgment, introducing uncontrolled variability. Additionally, functional and emotional impacts were assessed using self-reported measures, which are inherently susceptible to subjective bias.

Collectively, these limitations should be acknowledged when interpreting the findings, prospective assessment of LBP during pregnancy, and they underscore the need for future studies with multicenter designs, prospective assessment of LBP, inclusion of women with varying PA levels, and the use of validated and objective measurement tools.

## 5. Conclusions

This study shows that LBP during pregnancy is highly prevalent, even among PA women with a high educational level who had received a structured informational intervention on physical exercise and LBP prevention. Although pain intensity was generally mild to moderate, a substantial proportion of participants reported functional, emotional, and occupational limitations. The relatively low rate of medical consultation observed in this sample indicates that many women did not seek professional care for LBP, without allowing conclusions about the reasons underlying this behavior. These findings underscore the importance of improving awareness of LBP during pregnancy and ensuring access to appropriate information and support within prenatal care.

Regular PA was inversely associated with the presence of LBP, although the magnitude of this association was small and should not be interpreted as evidence of a clinically meaningful protective effect. Standing occupational position and a previous pregnancy-related LBP were identified as independent risk factors associated with the presence of LBP in the multivariable model; however, these associations should be interpreted with caution given the observational design and the borderline confidence interval for standing posture.

These findings suggest that incorporating systematic musculoskeletal assessment and promoting evidence-based prenatal exercise recommendations may help address LBP during pregnancy, although the potential impact of such strategies requires further evaluation in appropriately designed studies. Future research should explore the effectiveness of multidimensional preventive approaches and examine their influence on consultation patterns, functional outcomes, and women’s perceptions of LBP during pregnancy.

## Figures and Tables

**Table 1:** Incidence and temporal pattern of LBP during pregnancy.

Variable	Categories	Frequency (n)	Percentage (%)	*p* Value
Incidence of LBP (n = 147)				
LBP during pregnancy	Yes	95	64.6%	
	No	52	35.4%	
Trimester of onset and weekly frequency of LBP (n = 147)				
Trimester of onset (n = 95)	Third trimester	57	60.0%	<0.0001
	Second trimester	11	11.6%	
	First trimester	1	1.1%	
	Multitrimester * onset	26	27.3%	
Weekly frequency (n = 95)	>3 times/week	53	55.8%	*p* < 0.001
	Twice/week	21	22.1%	
	Three times/week	11	11.6%	
	Once/week	10	10.5%	

* Multitrimester onset includes cases originally classified as first–second, second–third, and first–second–third trimester.

**Table 2:** Clinical characteristics of LBP during pregnancy.

Variable	Categories	Frequency (n)	Percentage (%)	*p* Value
Chronobiology of LBP (subgroup with LBP; n = 95)				
Time of LBP	During the day	40	42.1%	<0.0001
	At night	27	28.4%	
	During the day and at night	17	17.9%	
	Other	11	11.6%	
Time of greatest LBP intensity	Afternoon	29	30.5%	
	Night	26	27.4%	
	Afternoon and night	18	18.9%	
	Other	22	23.2%	
Pattern, duration, radiation, and intensity of LBP (subgroup with LBP; n = 95)				
Type of LBP	Intermittent	65	68.4%	<0.001
	Continuous	30	31.6%	
Duration	Acute (<4 weeks)	48	50.5%	<0.001
	Subacute (4–12 weeks)	32	33.7%	
	Chronic (>12 weeks)	15	15.8%	
Irradiation pain	Yes	53	55.8%	
	No	42	44.2%	
Irradiation area (n = 53)	Gluteal region	19	35.8%	<0.001
	Leg	13	24.5%	
	Hip	8	15.1%	
	Other	13	24.6%	
LBP intensity by position (mean VAS)	Standing	4.9 ± 2.1	Moderate	
	Sitting	4.6 ± 2.1	Moderate	
	Lying supine	3.9 ± 2.5	Moderate	
	Lying on the side	2.7 ± 2.2	Mild	<0.001

VAS: Visual Analog Scale.

**Table 3:** Functional, emotional, and occupational impact, and therapeutic strategies for LBP during pregnancy.

Variable	Categories	Frequency (n)	Percentage (%)	*p* Value
Activities affected and aggravating factors (subgroup with LBP; n = 95)				
Activities affected	Work	25	26.3%	<0.001
	Sleep	16	16.8%	
	Walking	14	14.7%	
	Work + sleep	10	10.5%	
	Other	31	31.7%	
Aggravating factors	Sitting	36	37.9%	<0.001
	Standing	27	28.4%	
	Standing + household tasks	8	8.4%	
	Sitting + standing	8	8.4%	
	Other	16	16.9%	
Emotional and occupational impact (subgroup with LBP; n = 95)				
Impact on mood	Yes	50	52.6%	
	No	45	47.4%	
Work interruption	Yes	29	30.5%	<0.001
	No	66	69.5%	
Duration of sick leave due to LBP (only those who interrupted work; n = 29)				
	Mean	9.4 weeks		
	Range	0.5–28 weeks		
Therapeutic strategies perceived as effective (subgroup with LBP; n = 95)				
Effective treatments	Physical exercise only	32	33.7%	<0.001
	Exercise + massage	12	12.6%	
	Exercise + rest	12	12.6%	
	Other combinations	39	41.1%	

**Table 4:** Consultation for LBP during pregnancy and treatments recommended.

Variable	Categories	Frequency (n)	Percentage (%)	*p* Value
Consultation for LBP (n = 95)				
	Yes	42	44.2%	
	No	53	55.8%	
Total		95	100.0%	
Treatment recommendation (n = 42)				
	Yes	38	90.5%	<0.001
	No	4	9.5%	
Total		42	100.0%	
Type of treatment recommended (n = 42)				
	Physical exercise	24	57.1%	<0.001
	Pharmacological	6	14.3%	
	Pharmacological + Exercise	4	9.5%	
	None	4	9.5%	
	Other	4	9.5%	
Total		42	100.0%	

**Table 5:** Factors associated with the presence of LBP during pregnancy (multivariable logistic regression model).

Variable	Adjusted OR	95% CI	*p* Value
Maternal age (per 5 years)	0.89	0.72–1.10	0.28
Education level (university vs. secondary)	0.81	0.38–1.73	0.59
Prepregnancy BMI (per 5 kg/m^2^)	1.34	0.95–1.92	0.09
Parity (multiparous vs. primiparous)	1.76	0.84–3.68	0.13
Occupational position:			
Standing position (vs. sitting)	2.14	1.00–4.55	0.047 *
Mixed position (vs. sitting)	1.58	0.78–3.18	0.20
Working hours/day (per 1 h)	1.09	0.94–1.26	0.24
PA during pregnancy (per 500 MET·min/week)	0.91	0.83–0.99	0.032 *
Prepregnancy PA (per 500 MET·min/week)	0.97	0.89–1.06	0.50
Previous pregnancy-related LBP (yes vs. no)	2.89	1.12–7.48	0.029 *

OR: odds ratio; CI: confidence interval; MET: metabolic equivalent of task; *: *p* < 0.05.

## Data Availability

The data presented in this study are available on request the corresponding author due to legal and ethical reasons.
